# Stem Cell Niche Dynamics: From Homeostasis to Carcinogenesis

**DOI:** 10.1155/2012/367567

**Published:** 2012-02-09

**Authors:** Kevin S. Tieu, Ryan S. Tieu, Julian A. Martinez-Agosto, Mary E. Sehl

**Affiliations:** ^1^Computational and Systems Biology Interdepartmental Program, School of Medicine, University of California, Los Angeles, CA 90095, USA; ^2^Department of Human Genetics, School of Medicine, University of California, Los Angeles, CA 90095, USA; ^3^Division of Hematology-Oncology, Department of Medicine, School of Medicine, University of California, Los Angeles, P.O. Box 957059, Suite 2333 PVUB, Los Angeles, CA 90095-7059, USA

## Abstract

The stem cell microenvironment is involved in regulating the fate of the stem cell with respect to self-renewal, quiescence, and differentiation. Mathematical models are helpful in understanding how key pathways regulate the dynamics of stem cell maintenance and homeostasis. This tight regulation and maintenance of stem cell number is thought to break down during carcinogenesis. As a result, the stem cell niche has become a novel target of cancer therapeutics. Developing a quantitative understanding of the regulatory pathways that guide stem cell behavior will be vital to understanding how these systems change under conditions of stress, inflammation, and cancer initiation. Predictions from mathematical modeling can be used as a clinical tool to guide therapy design. We present a survey of mathematical models used to study stem cell population dynamics and stem cell niche regulation, both in the hematopoietic system and other tissues. Highlighting the quantitative aspects of stem cell biology, we describe compelling questions that can be addressed with modeling. Finally, we discuss experimental systems, most notably *Drosophila*, that can best be used to validate mathematical predictions.

## 1. Introduction

The hematopoietic stem cell niche is an important regulator of stem cell fate. There are complex signaling pathways, such as Notch, Wnt, and Hedgehog, that carefully regulate stem cell renewal, differentiation, and quiescence [1–3]. Mathematical models can be useful in studying the dynamics of stem cell maintenance. Quantitative models can provide information about cell population dynamics, regulatory feedback of interacting networks, and spatial considerations related to the structural relationships between stem cells and their progeny with cells of the microenvironment.

Errors in stem cell division rate or in the balance between self-renewal and differentiation may result in tissue overgrowth or depletion [4]. One novel target of cancer therapeutics is the stem cell niche [5, 6]. Stem cell niche signaling inhibitors are being designed with the idea that regulatory signals that are active in stem cell niche homeostasis may go awry during carcinogenesis [6–8]. Understanding the biology and dynamics of stem cell behavior under normal conditions and examining how the dynamics change under conditions of stress is essential to our understanding of how these mechanisms might change during carcinogenesis.

Mathematical and physical models have been used to study stem cell population dynamics and the regulation of stem cell fate through niche signaling with great success. We present a review of quantitative approaches to understanding stem cell niche signaling in the hematopoietic system, as well as in other tissues under conditions of homeostasis and carcinogenesis. We explain the benefits of mathematical models in advancing our understanding of the mechanisms of regulation of stem cell fate and how this regulation changes in cancer development. We describe models that incorporate spatial aspects of the regulation of asymmetric division and compare normal conditions to carcinogenesis. We highlight the synergistic relationship between mathematical predictions and experimental validation and illustrate *Drosophila* as a model system for quantitative studies of the stem cell niche. Finally, we address the potential for mathematical models to predict and optimize therapies targeting the stem cell niche.

## 2. Quantitative Aspects of the Hematopoietic Stem Cell Niche

Hematopoietic stem cells (HSCs) are a dynamically well characterized stem cell population. The hematopoietic system was the first system in which multipotency, or the ability for a single HSC to regenerate all of the different cell types within the tissue, was described. A second defining characteristic for stem cells, self-renewal, has also been demonstrated in HSCs. Self-renewal is the ability of the HSC to generate a genetically identical copy of itself during cell division. This can occur asymmetrically, giving rise to one identical copy and one partially differentiated daughter cell, or symmetrically, giving rise to two identical copies of itself. Single HSCs have been shown to be self-renewing, multipotent, and to cycle with slow kinetics. Extrapolation from feline and murine data suggests a symmetric birth rate for human HSCs of once every 42 weeks [9]. Quiescence, the state of not dividing, allows HSCs to avoid mutation accumulation and contributes to their long lifespan. In contrast to senescence, where the cell loses its ability to undergo division, a cell can reawaken from the state of quiescence to an activated state where it can again undergo self-renewal.

The stem cell microenvironment regulates stem cell self-renewal, differentiation, quiescence, and activation. While little in situ information is known about the anatomy and structural relationships of the hematopoietic stem cell and its niche, there is a growing amount of experimental information about the behavior of signaling systems that govern HSC fate.

Population dynamics models have been successfully used to model the human hematopoietic system in both health and disease [9–17]. Using stochastic and deterministic models, significant progress has been made in understanding the dynamics of cancer initiation and progression [18, 19] and the sequential order of mutation accumulation [20]. Mathematical models have also been useful in modeling leukemic stem cell and progenitor population changes in response to therapy and the development of resistance [14].

An ongoing debate in hematopoietic stem cell biology concerns how much variability exists in hematopoietic stem cell fate [21]. Stochastic models have been used to study the dynamics of clonal repopulation [22] following hematopoietic stem cell transplant. In these models, trajectories of hematopoietic stem cell counts as well as progenitor and differentiated cell counts are generated and compared with observed cell counts. Rates of self-renewal, differentiation, and elimination of cells are estimated. Stochastic trajectories are found to match experimental results. These models predict that hematopoiesis is probabilistic in nature and that clonal dominance can occur by chance. These models could be enhanced by examining regulators of stem cell fate by the microenvironment. Stochastic simulation can be used to incorporate elements of the stem cell niche, such as surrounding stromal cells and signaling pathways, and model cell-cell and cell-environment interactions. These models could identify regulators of stem cell fate and explore the dynamics of this regulation.

Chronic myelogenous leukemia (CML) represents a nice system to quantitatively study hematopoietic stem cell and progenitor dynamics. CML is the first malignancy recognized as a stem cell disorder. The translocation t(9;22) is present in leukemic stem cells, multipotent progenitors, and their progeny of the myeloid lineage. This translocation leads to transcription of the BCR-ABL fusion oncogene which is thought to regulate cell survival. Therapy inhibiting BCR-ABL is one of the first examples where chronic administration of a molecularly targeted therapy has led to a dramatic clinical response. This response is observed in all phases of the disease.

Mathematical models have been used to demonstrate that leukemic stem cells are not targeted by imatinib therapy [14], and that successful therapy must target leukemic stem cells [12]. Other models have highlighted the importance of leukemic stem cell quiescence as a mechanism leading to therapeutic resistance [13].

In a study of chronic myelogenous leukemia under targeted therapy, Michor et al. [14] describe the dynamics of leukemic stem cells and the development of resistance using a Moran process model. Based on calculated rates of death and differentiation using data of biphasic decline of BCR-ABL transcripts, they conclude that the leukemic stem cell compartment is not sensitive to therapy. An alternative explanation is provided by Komarova and Wodarz [13], using a stochastic model in which quiescence and reactivation of leukemic stem cells are considered. In this work, the biphasic decline of BCR-ABL transcripts is explained by the elimination of active leukemic stem cells, followed by the slower elimination of quiescent leukemic stem cells following their reactivation. This study offers hope that targeted therapy, used in combination with potential therapies that lead to activation of quiescent cells, could eradicate the stem cell-like compartment of a tumor. These models could be expanded by modeling the contribution of the microenvironment that regulates quiescence and activation of stem cells. Validation of these models will require experimental determination of rates of quiescence and reactivation to obtain accurate parameters for modeling.

Birth-death process models have been used to study extinction of leukemic and normal hematopoietic stem cells under therapy targeting leukemic stem cells. These models conclude that the killing efficiency of a therapy is a major determinant of the mean time to extinction of leukemic stem cells (optimal duration), while the selectivity of a therapy predicts the average number of normal hematopoietic stem cells at the time of leukemic stem cell extinction (safety) [23]. Incorporating quiescence in these models reveals that a successful therapy needs to target both active and quiescent leukemic stem cells.

We extended this model to consider combination of therapy targeting leukemic stem cells, and their niche was considered using stochastic simulation. Because stem cell self-renewal is expected to decrease with Wnt-inhibitor therapy, we modeled the addition of niche-targeted therapy as a decrease in birth rates of leukemic stem cells. We found that this combination can be effective in eliminating the leukemic stem cell compartment, even when the effects of BCR-ABL-targeted therapy on stem cells are modest. We anticipate that extension of these models to include regulatory feedback of the stem cell microenvironment using stochastic reaction kinetic methods would be very helpful in modeling dynamics of niche-targeted therapies.

The hematopoietic stem cell niche has been studied in the healthy hematopoietic system. A model based on self-organizing principles demonstrates the importance of asymmetry in determining stem cell fate and concludes that stem cell fate is only predictable in describing populations rather than individual cellular fates [24]. Deterministic models are useful in simulating proliferation and differentiation of all populations comprising the stem cell niche [25]. These studies conclude that kinetics are highly variable because of the relatively small number of cells proliferating and differentiating in the niche. Experimental studies have examined the role of Wnt signaling in regulation of normal hematopoietic regeneration [26]. We expect the combination of mathematical modeling with experimental validation to prove useful in modeling the pathways under normal conditions and dysregulation of these pathways during stress, inflammation, and carcinogenesis.

**Figure 1:**
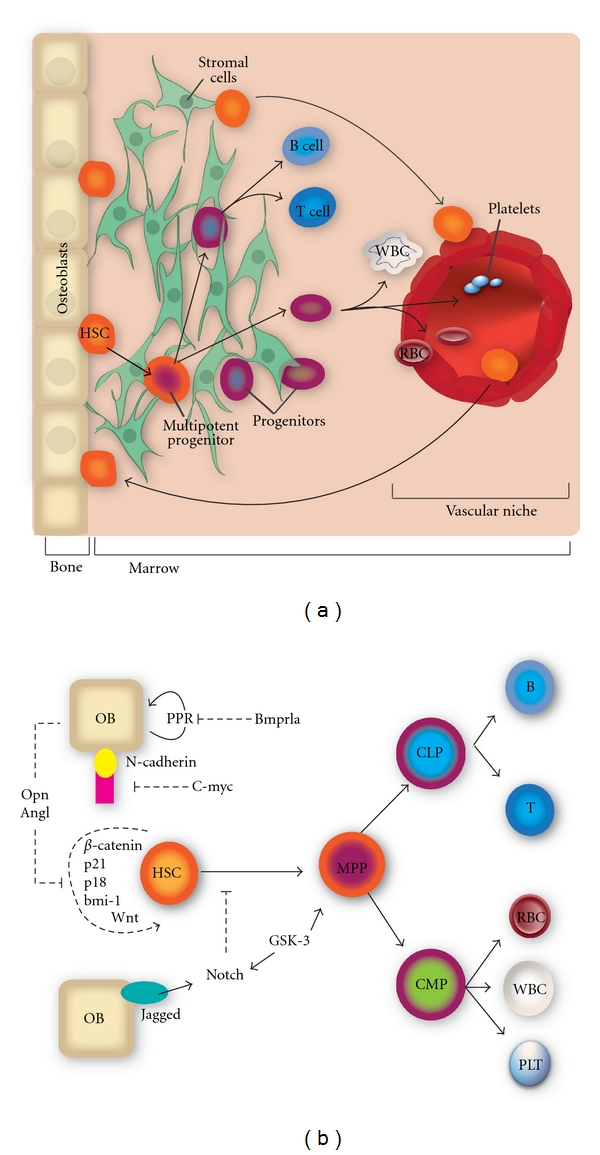
Quantitative aspects of the hematopoietic stem cell (HSC) niche. The left panel provides a structural picture of the niche, while the right panel shows a schematic representation of a mathematical model for the regulation of hematopoietic stem cell fate. The model incorporates population counts and signaling pathways that may play a role in regulating stem cell population dynamics. Cellular populations comprising the bone and vascular niches include osteoblasts (OBs), endothelial cells, HSCs, multipotent progenitors (MPPs), common myeloid progenitors (CMPs), common lymphoid progenitors(CLPs), and differentiated cells. Signaling from Wnt, *β*-catenin, p21, p18, and bmi-1 regulate self-renewal, while Notch and GSK3 feedback from progenitors inhibit differentiation that usually accompanies self-renewal. Signaling from osteoblasts includes osteopontin (Opn) expression that inhibits HSC self-renewal, parathyroid hormone-related protein (PPR) which increases HSCs, N-cadherin which binds *β*-catenin, and Tie2/angiopoietin which regulates quiescence.

Figure 1 describes the elements of the HSC niche and an accompanying schematic representation of a mathematical model of the niche. The model captures the key regulatory components of niche dynamics, including cell population sizes and the signaling pathways that regulate them.

## 3. *Drosophila* as a Classic Model System

*Drosophila* represents an excellent model system to study stem cells, their microenvironment, and the tight regulation of homeostasis through different signaling pathways. The male *Drosophila* germ line population is a classic system used to study properties of the stem cell niche [27, 28]. The power of this model includes the ability to quantify cell populations over time, the relatively quick repletion of lost cells with newly differentiated cells, and the ability to experimentally observe spatial effects. These quantitative aspects, as well as its simple, well-characterized lineages, make the *Drosophila* experimental system ideally suited for the development and validation of mathematical modeling. Finally, vertebrate and invertebrate digestive systems show extensive similarities in their developments, cellular makeup, and genetic control [29].

Mathematical and physical models have been used to study regulation of stem cell fate through niche signaling in the *Drosophila* blood and midgut [30], as well as in the *Drosophila* eye [31] and the *Drosophila* embryo [32], with great success. Studies of the stem cell niche in model systems such as *Drosophila* have revealed adhesive interactions, cell cycle modifications, and intercellular signals that operate to control stem cell behavior [4, 33]. These interactions have been studied quantitatively. For example, Wnt and Notch play pivotal roles in stem cell regulation in the *Drosophila* intestine [30, 34]. In addition, the APC gene has been shown to regulate *Drosophila* intestinal stem cell proliferation [35]. APC is well known to play a role in human colon carcinogenesis, and mathematical models have shown that stem cell proliferation leads to colon tumor formation in humans [36, 37].

The spatially patterned self-renewal and differentiation of stem cells has been extensively studied in *Drosophila* embryonic studies of development [32, 38–40]. The spatial orientation of stem cells has been visualized in *Drosophila* brain and testes and has recently been shown to be of great importance in experimental models of neuroblastoma growth in *Drosophila* [41]. We anticipate that the combination of spatial effects simulation and direct visualization of the *Drosophila* midgut through experiment will advance our understanding of the interaction of alterations in signaling pathways and spatial effects in carcinogenesis. 

## 4. Extension to Inflammation and Carcinogenesis across Tissues

**Figure 2:**
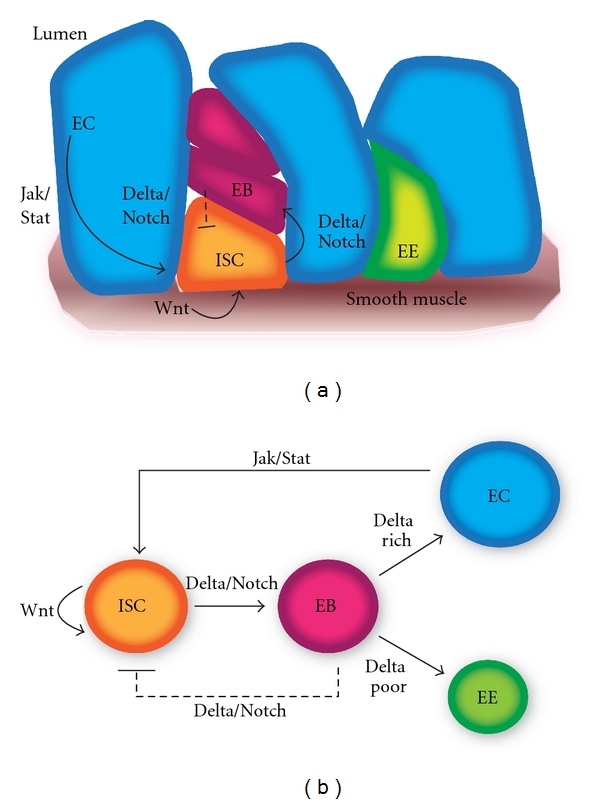
Structural and dynamic aspects of the *Drosophila* intestinal stem cell (ISC) niche. The left panel shows a structural picture of the *Drosophila* intestine, while the right panel reveals population and regulatory elements of a mathematical model for ISC regulation. Populations of the intestinal stem cell niche in the *Drosophila* include ISCs, enteroblasts (EBs), enteroendocrine cells (EE), and enterocytes (ECs). Wnt signaling from underlying smooth muscle and Notch feedback from EB regulate ISC self-renewal, while Jak/Stat feedback from damaged ECs increases ISC self-renewal.

**Figure 3:**
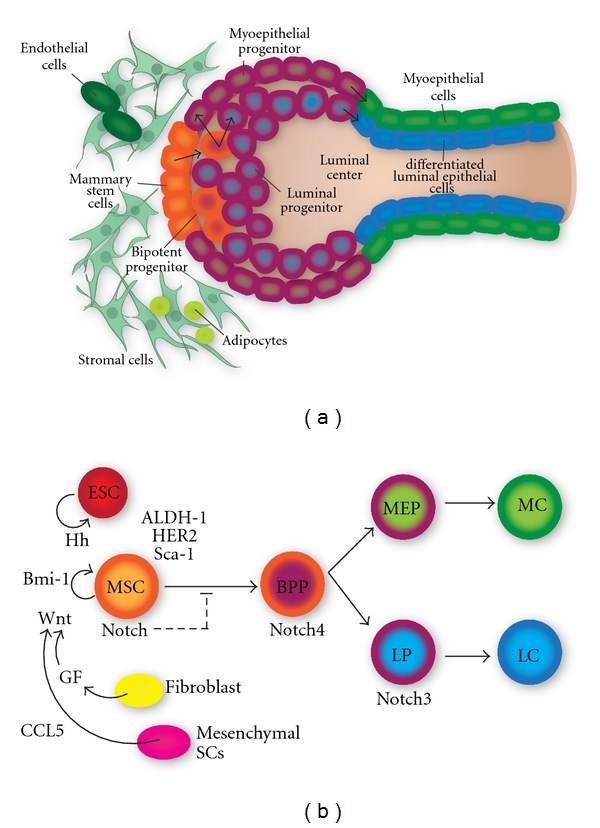
Model of the breast stem cell niche including structural elements (left panel) and mathematical model (right panel). Key populations of the mammary stem cell niche include mammary stem cells (MSCs), mesenchymal stem cells, endothelial stem cells (ESCs), bipotent progenitor (BPP), luminal progenitor (LP), myoepithelial progenitor (MEP), myoepithelial cells (MCs), luminal epithelial cells (LCs), and stromal cells. Wnt, Notch, and Hedgehog (Hh) signaling play a role in MSC self-renewal. Regulatory signals from growth factors (GFs) secreted by fibroblasts and CCL signaling from mesenchymal stem cells also regulate MSC fate.

Unifying features of stem cell niche regulation are observed across tissues and across organisms [42, 43]. Figures 1, 2, and 3 compare the structural and signaling elements of the stem cell niche across the hematopoietic, intestine, and breast tissues. While little is known about the structural orientation of the human hematopoietic stem cell niche 1, much has been learned about the signaling pathways in both the bone and vasculature that regulate HSC fate. Osteoblasts (OBs) express osteopontin which negatively regulates HSC proliferation. Tie2/angiopoietin signaling regulates HSC anchorage and quiescence, and adherence to osteoblasts. HSCs and OBs are increased via the parathyroid hormone-related protein receptor (PPR) expressed in OBs. OBs express N-cadherin which forms a beta-catenin adherens complex with HSCs. C-myc negatively regulates N-cadherin in differentiating HSCs and promotes differentiation and displacement from the endosteum. OBs express Jagged-1, a Notch receptor that when bound inhibits differentiation that usually accompanies Wnt-induced HSC proliferation. GSK-3 activity enhances HSC progenitor activity and may control asymmetric cell division by modulating Notch and Wnt signaling pathways.

Figure 2 depicts the intestinal stem cell niche of *Drosophila*. Here, we see four key cellular populations: intestinal stem cells (ISCs), enteroblasts (EBs), enterocytes (ECs), and enteroendocrine (EE) cells. It has been previously established that ISCs can self-renew under the influence of the Wnt signaling pathway [44] and can asymmetrically divide giving rise to one partially differentiated EB cell and one ISC, under the influence of the Delta/Notch signaling pathway. EBs can then differentiate into either EC cells or EE cells. There is feedback from the EB population to the ISC population, which inhibits self-renewal and differentiation, in order to maintain stable population sizes under the normal conditions of homeostasis [45]. The EC population also interacts with the ISC population via Jak/Stat signaling feedback, which increases self-renewal and differentiation, in conditions when EC loss occurs [45]. 

Finally, both structural and signaling aspects of the breast stem cell niche are shown in Figure 3. The hedgehog (Hh) pathway is required for normal development of the mammary gland and regulates self-renewal of human mammary stem cells (MSCs) [46–48]. Hh also targets endothelial cells and induces angiogenesis by promoting endothelial progenitor proliferation and migration. Wnt signaling regulates proliferation, apoptosis, and differentiation and maintains stem cells in a self-renewing state. Notch promotes self-renewal in normal mammary stem cells [46, 49]. Notch3 is expressed in epithelial progenitors, and Notch4 is expressed in bipotent progenitors. Markers of mammary stem cells include ALDH1 expression, and Sca-1. There is a significant correlation between expression of ALDH1 and HER2 overexpression [50].

The common signaling pathways that control stem cell self-renewal in these pathways, such as Notch, Wnt, and Hedgehog, are known to play a role in carcinogenesis [2, 41]. A growing body of evidence from a variety of solid tumors suggests that the first carcinogenic cell within a tumor possesses stem cell properties, including self-renewal, increased cell survival, limitless replicative potential, and the ability to produce differentiating cells [51–60]. However, it is unclear whether accumulation of mutations within a tumor cell with stem cell properties or extrinsic factors originating in the tumor microenvironment drive tumor progression [61, 62]. Understanding niche signaling pathways under normal conditions, and in response to inflammation and stress response, is vital to understanding how they may go awry in carcinogenesis.

The known link between inflammation and cancer may involve the regulation of stem cell fate by inflammatory cytokines [63]. IL-1, IL-6, and IL-8 are known to activate Stat3/NF-*κ*B pathways in tumor and stromal cells. Positive feedback loops are formed involving further cytokine production which can drive cancer stem cell self-renewal [63]. These networks can be nicely modeled using stochastic reaction kinetics. Predictions from these models could be used to guide therapy design.

Dysregulation of normal homeostatic processes in the human hematopoietic stem cell niche may lead to enhanced self-renewal and proliferation, enforced quiescence, and resistance to chemotherapeutic agents. Leukemic stem cells have been shown to infiltrate the normal HSC niche by direct invasion or secretion of substances such as stem cell factor [6]. Leukemic stem cells may also exhibit dysregulated homing and engraftment, leading to alternative niche formation [6]. Future mathematical models of leukemic stem cell dynamics should take into account the stem cell niche.

Cytokine/Jak/Stat signaling has recently been shown to mediate regeneration and response to stress in the *Drosophila* midgut [45, 64]. Mathematical models of proliferation and differentiation of *Drosophila* intestinal stem cells have examined the dynamics of Wnt and Notch signaling [30], but have not yet examined the feedback of Jak/Stat signaling from the differentiated enterocytes to intestinal stem cells. Mathematical models of the human intestinal stem cell niche have shown that dysregulated colonic crypt dynamics cases stem cell overpopulation and initiate colon cancer [36]. Symmetric division of cancer stem cells has been shown to be a key mechanism of tumor growth to target in therapeutic approaches [37].

In mammalian systems, MyD88 and RAS signaling have been shown to lead to mouse and human cell transformation [65]. These signaling pathways are known to be involved with inflammation and also play a direct role in cell cycle control. The link between inflammation and carcinogenesis needs to be studied quantitatively.

Alterations in Wnt signaling contribute to excess proliferation of mammary progenitor cells leading to cancer [66]. Unregulated Notch signaling in the mouse mammary gland leads to tumor formation. Increased expression of Notch in ductal carcinoma is associated with shorter time to recurrence [67]. Breast density is an important risk factor for breast tumor development [68], suggesting a role of the stem cell microenvironment in carcinogenesis. Growth factors secreted by fibroblasts influence mammary stem cell behavior. Endothelial cell and adipocytes may also influence stem cell behavior. CCL5 secretion by mesenchymal stem cells influences stem cell self-renewal. Alterations in Notch signaling are thought to play a role in breast cancer development.

Combination of theory and experiment has shed light on stromal-tumor interactions in the human breast [69]. In the breast, ductal cells secrete TGF-beta and fibroblasts secrete EGF. During carcinogenesis, TGF-beta then transforms fibroblasts into myofibroblasts, which in turn secrete higher EGF. Mathematical modeling has shown that this feedback system increases proliferation of tumor cells, and theoretical results match experimental validation well.

Mathematical models have also shed light on the interactions between the stem and nonstem compartments of solid tumors and their effects on the heterogeneous growth of solid tumors. These models show that apoptosis of nonstem cells paradoxically leads to tumor growth and progression [70, 71].

Cancer cell plasticity is an important consideration in the study cancer stem-like cells in oncology. The finding that nonstem cells can dedifferentiate to a stem-like state in mammary cell lines [72] has important implications in defining cancer stem-like cells and identifying therapies to target them. Markov models have recently proven very helpful in calculating rates of dedifferentiation of mammary epithelial cells to stem-like cells [73]. Consideration of microenvironmental signaling that regulates these transitions will greatly enhance these models and their predictions.

## 5. Spatial Considerations in Modeling Stem Cell Regulation

**Figure 4:**
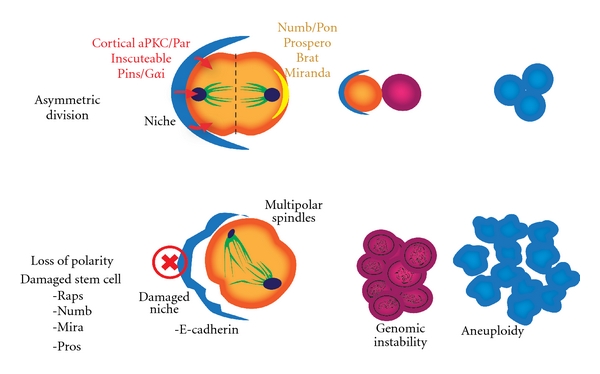
Stem cell polarity: homeostasis and dysregulation. Regulation of asymmetric division in the stem cell niche. The left panel represents spatial regulation of normal homeostasis, while the right panel demonstrates the loss of this asymmetry during carcinogenesis.

Spindle orientation is well known to play a role in stem cell fate [74]. Asymmetric division is regulated by maintaining the stem cell orientation, and this is regulated by its spatial relationship with the cells of the niche. Induction of brain tumor growth has been demonstrated by altering stem-cell asymmetric division in *Drosophila* melanogaster [41]. Loss of cell polarity and cancer are tightly correlated [4]. In stem cells, loss of polarity leads to impairment of asymmetric cell division, altering cell fates, rendering daughter cells unable to respond to the mechanisms that control proliferation. The tumor suppressor p53 regulates polarity of self-renewing divisions in mammary stem cells [75].  Figure 4 displays regulation of stem cell asymmetric division under normal homeostatic conditions and the loss of this regulation during carcinogenesis. Labeling of template strands in stem cells of small intestine crypts using tritiated thymidine reveals selective retention of parental DNA strands and loss of newly synthesized strands during stem cell division [76]. This mechanism provides the stem cell with protection from DNA replication errors during asymmetric division. Loss of asymmetric division may lead to loss of this protection against chromosomal instability.

Mathematical models that allow for the inclusion of spatial effects are necessary in order to study this loss of asymmetry in the stem cell and its relation to carcinogenesis. Classic models of spatial effects on development in *Drosophila* have examined reaction diffusion equations [38, 39]. While multiscale models are more recently being used to study complex biologic systems and their genetic regulation, most of the methods used assume a well-stirred system and have not allowed for consideration of spatial effects until recently. Incorporating a spatial component into stochastic simulation methods is an exciting frontier in stochastic reaction kinetics [77, 78]. A stochastic reaction-diffusion equation is used in place of the chemical master equation and is sampled in the stochastic simulation. These methods have been shown to be successful in modeling spatial effects in genetic regulatory networks [78].

## 6. Conclusions

Mathematical models have proven useful in characterizing stem cell and progenitor cell population dynamics, and in understanding the interacting components of the stem cell niche. Identifying quantitative characteristics of the stem cell microenvironment that are generalizable across tissues, as well as those distinct to each system, will be necessary to help define the emerging concept of the stem cell niche. Modeling the components of the stem cell niche and their interactions will advance our understanding of the tight regulation of stem cell fate. In turn, it will allow us to predict and validate responses to stress, inflammation, and carcinogenesis. In addition to quantifying population distributions and feedback networks, it will be necessary and informative to incorporate spatial aspects that govern asymmetric versus symmetric stem cell self-renewal. We expect that the combination of predictive modeling and experimental validation will prove useful in our understanding of the regulatory components of stem cell maintenance and the changes that occur in response to treatments designed to target the stem cell niche.
